# *Erema po otjindjumba*? Highlighting cultural models and knowledge gaps of malaria in rural Namibian pastoralists

**DOI:** 10.1186/s12936-025-05382-x

**Published:** 2025-05-07

**Authors:** Sean Prall, Aparicio Lopes

**Affiliations:** 1 Department of Anthropology, University of California, Los Angeles, USA; 2 OnePencil Namibia, Opuwo, Namibia

**Keywords:** Malaria, Indigenous pastoralists, Namibia, Cultural models, Qualitative research

## Abstract

**Background:**

As Namibia attempts to eradicate locally transmitted cases of malaria, epidemiological strategies, interventions, and outreach require a sound understanding of indigenous knowledge and practice. Research describing local explanatory models of disease can be of value in these efforts by elucidating how disease is interpreted and treated. To understand how perceptions of malaria infection and treatment may influence health-seeking behaviour, cultural models of the disease were explored in two ethnic groups in rural northwest Namibia.

**Methods:**

Mixed-sex focus groups of 4–8 individuals were conducted in the Kunene region of Namibia. All participants were either Himba or Herero and lived between 14 and 57 km of the regional town centre of Opuwo. Discussion prompts were designed to assess knowledge, beliefs, and norms about malaria, including causes, symptoms, treatment, and prevention.

**Results:**

Focus groups reported universal difficulty in discrimination between malaria and respiratory infections, the former of which was often only diagnosed at the hospital. Some recognized mosquitoes as the source of malaria, particularly the more formally educated Herero, but all also reported other causes. Notably these causes, including dietary and temperature-based origins, were considered unavoidable. Himba and Herero believed that malaria was infectious person-to-person and incorrectly believed that malaria was most common during the wintertime. Both groups also relied on a number of traditional remedies to alleviate symptoms, which were used as primary treatment, with formal healthcare treatment typically only sought when the illness progressed.

**Conclusions:**

These results highlight significant differences between local cultural models and biomedical ones that could be detrimental to malaria eradication efforts. Kunene pastoralists have limited understanding of the causes of malaria, and beliefs about environmental and dietary causes may undermine attempts at prevention. Seeking healthcare solutions to malaria was normative, but secondary to use of at home traditional remedies. These findings indicate public health outreach and information campaigns are needed, particularly in rural groups with less formal education.

## Background

Malaria is endemic to several northern regions of Namibia, including the rural Kunene, which is home to several large pastoralist groups. In the last 20 years, the Namibian government has made significant efforts to reduce malaria infection and transmission in an effort to eradicate the disease. Increased use of rapid diagnostic tests, indoor residual spraying (IRS), artemisinin-based combination therapy (ACT), and use of long-lasting insecticidal nets (LLINs), has led to a 97% reduction in malaria cases between 2001 and 2011 [1, 2]. The newly instituted National Malaria Elimination Strategic Plan 2023–2027 aspires to reach zero locally contracted malaria cases by 2027 [3]. While major progress has been made towards this goal, numerous operational, technical, and resource allocation problems remain in reducing vector populations, reducing transmission, diagnosis, treatment, and surveillance of cases [1]. Locally transmitted case counts actually rose 12.4% between 2015 and 2022, even as the country saw a reduction in malaria mortality [4]. Unfortunately, areas of northern Namibia remain stubbornly malaria endemic, with a previous eradication goal missed in 2020.

Difficulty at reducing malaria cases in northern Namibia is due to a number of factors, including frequent travel across the border to Angola [5]. In response, the Trans-Kunene Malaria Initiative (TKMI), an agreement between Namibia and Angola to collaborate in data sharing and support in initiatives to reduce malaria transmission, was signed in 2011 [6]. These efforts appeared to have paid off, as interventions that were part of the TKMI resulted in reduced odds of child fever in both Namibia and Angola [7]. However, other factors, like SES, demographics, housing characteristics including use of open structures and traditional home construction, rainfall and vegetation, and pastoralism continue to be associated with risk of infection in the region [5, 8].

These features may be particularly resonant for the large number of indigenous populations in the Kunene, most of whom are traditional rural living communities relying on agro-pastoralism for subsistence and often residing some distance from clinics or hospitals. As Namibia attempts to achieve elimination, adequate surveillance, timely testing, and treatment of these populations is vital. Mobile pastoralists tend to have lower IRS coverage, and this coverage is less effective where people have open structures and often sleep outside [1]. Early testing and treatment is essential to controlling and reducing malaria infections, as infected individuals who delay treatment are likely to experience longer infectious periods [9, 10]. This is particularly challenging in rural communities where distance to clinics is greater and people are more mobile.

Controlling and ultimately eliminating a disease like malaria does not just rely on health infrastructure, but also on local knowledge and beliefs about disease, appropriate remedies, and norms and perceptions regarding clinical treatment. Diseases where the local populace refuses testing and treatment may be more difficult to manage. As such, understanding local cultural models of malaria illness and treatment is crucial to the goal of eliminating malaria in Namibia.

Local culturally derived models of illness are common across subsistence based small-scale societies and in Africa [11, 12]. These models shape how illness is understood, problematized, and treated. They are particularly important to understand when they conflict with prescribed biomedical practices or treatments [13]. For example, researchers working with the Acholi of Uganda found that they used multiple explanatory or cultural models to understand the outbreak of Ebola haemorrhagic fever (EHF) [14]. Some of these beliefs were consistent with biomedical recommendations, such as quarantine. However, others, such as traditional funeral rituals, increased the spread of EHF. Similarly, local beliefs about contagion and immunity shaped mask-wearing and physical distancing in communities in the Philippines during the COVID-19 pandemic, raising the prospect of moral contagion and viewing people as contagious by association with particular places [15]. More broadly, Napier et al*.*, in a special The Lancet Commissions report [16], stated that “in all cultural setting the need to understand the relation between culture and health, especially the cultural factors that affect health-improving behaviors, is now crucial.”

In the Kunene, little has been published about local cultural and explanatory models of disease. The region is home to numerous indigenous groups, primary Himba, Herero, and several other closely related agropastoral groups. These populations share language and some similar cultural features [17], including several practices which make them at greater risk for malaria infection. These groups, particularly when living in rural areas, tend to be of low socio-economic status. Himba also live across the Kunene River in southern Angola, and frequently travel across the international border to visit friends and family and engage in other cultural activities. Many live in traditional dwellings and spend significant time engaged in horticultural activities and herding livestock in the bush. These traits are associated with higher rates of malaria infection, placing these indigenous populations at greater risk [8]. As Namibia attempts to achieve zero locally contracted malaria cases, focusing on these at-risk groups will become crucial. This is particularly true as the number of malaria cases dwindle, making traditional control interventions less effective, and requiring more specialized population-specific approaches [18]. Effective strategies must include consideration of how local explanatory models of disease impact testing and treatment to make progress. This will be particularly important for mobile, indigenous populations like the ones studied here.

To better understand beliefs and behaviors about malaria among at-risk indigenous populations in rural northern Namibia, this study aimed to 1) explore local cultural models of malaria, and 2) examine norms of treatment, use of traditional medicines, and feelings and experiences at health care facilities. To address these aims, mixed-sex focus groups of Himba and Herero living in rural and peri-urban contexts were conducted. These qualitative results were interpreted with an eye towards exploring knowledge gaps of malaria aetiology, treatment, and prevention, and results used to provide suggestions for future public health outreach efforts.

## Methods

### Study site

This qualitative study was conducted in rural communities outside of the regional town centre, and regional hospital, of Opuwo in the Kunene region of northern Namibia. Known Himba and Herero communities outside of Opuwo were opportunistically sampled. Average community distance from town was 30 km (min = 14.5 km, max = 57 km), including several that were more than 10 km from any main gravel road. The region is dry and arid, and all indigenous groups in the area typically practice some form of mixed subsistence that includes pastoralism and small scale horticulture, although local livelihoods have been heavily impacted by recent drought [19]. The region has high levels of poverty and inequality, and low human development scores [20, 21]. Residents of the area also suffer from poor access to clinics and hospitals [22]. Healthcare access problems are exacerbated by cost of travel and costs of medical fees as well as a high doctor-to-patient ratio [22–24]. The vast majority of malaria cases are reported in Opuwo [1], which is considered to be a region of moderate transmission risk [25].

Himba live in traditional dwellings made from mud and cow dung, with a central fire and a single opening to the outside [26]. Other times they may sleep out of doors when traveling or spending time in the bush herding livestock. They tend to have very low levels of formal education, and typically dress in traditional fashion, both features that make them prone to discrimination in healthcare settings [27]. Unlike the more market-integrated Herero, Himba are largely excluded from the cash economy, due to decades of social and economic marginalization [28], and are of low socio-economic status compared to populations living in or near the regional town centre of Opuwo. Kunene Herero, by contrast, are more market integrated, and many live closer to town. Many Herero engage in wage labour and tend to be more educated than their Himba counterparts. Herero households tend to rely more on non-traditional building materials like bricks and sheet-metal. They also typically dress in a more western fashion. Nevertheless, Herero often maintain connections to rural villages, pastoralism, and emphasize traditional practices like matrilateral inheritance.

### Data collection procedures and analysis

Across 6 communities 4 Himba and 3 Herero mixed sex focus groups were conducted, each between 4 and 8 individuals following best practices [29, 30]. These methods have been used extensively elsewhere to understand explanatory models of illness in other contexts [e.g. 14, 31, 32]. Focus group questions and discussion centred on knowledge and practices regarding malaria, with a particular emphasis on how Himba and Herero define and understand this illness. Discussions included queries about how malaria is treated, including traditional medicines and the pursuit of treatment at clinics and the local hospital. All focus group interviews were conducted in the Otjiherero, a Southwest Bantu language in Guthrie zone R (code R.311). Focus groups were instantaneously translated, and audio recordings used to create transcriptions based on this translation. When possible, relevant local terms were included in transcriptions. Each focus group took between 60 and 90 min. Analysis of focus group transcriptions was conducted using the open-source qualitative package Taguette [33]. Transcriptions were thematically coded, with initial codes derived based on the goal of explaining cultural interpretations of malaria, and iteratively updated during the analysis phase following best practices [34]. An interview guide and COREQ checklist is available elsewhere [35].

## Results

### Difficulty differentiating between respiratory illness and malaria

In both Himba and Herero focus groups, participants reported difficulty differentiating between respiratory illness and malaria. This can be attributed to three factors; naming convention, reported similarities in symptoms, and the necessity of medical diagnosis at a hospital for malaria. First, the word influenza—or *otjindjumba* in Otjiherero—is often used interchangeably with malaria and Himba and Herero participants frequently used both in casual conversation about malaria. Others describe malaria (and COVID-19) as a type of *otjindjumba* with special properties. Malaria is typically referred to as just malaria or sometimes referred to as *erema*. The word *erema* is described as an older Otjiherero word which has since largely been replaced by the modern term *malaria*, and not all participants knew this word. As one senior Himba man defined it:

*Erema means a disease which is strong and puts you in bed, takes all your strength away and puts you in bed. It disables you.*

This same man noted that he first heard the word “malaria” at a rural health clinic in the 1970 s. Prior to that *erema* or *otjindjumba* was used.

When queried, participants report some specific symptoms, which can partially aid in differentiation. Malaria is associated with fever and weakness. Many participants emphasized malaria is associated with periods of feeling hot and cold, and being unable to keep warm. For example:

*When the person has erema, they will bring blankets and you cover yourself with blankets because its hot and you are in the sun, but your body is shivering. You are cold, you’re freezing, they cover you with lots of blankets in the sun but you are still shivering and feeling cold.*

Another Herero man described malaria as a type of flu, noting that “*Malaria is an otjindjumba that makes one weak and cold*.”

Other symptoms emphasized include vomiting, weakness, diarrhoea, loss of appetite, headaches, and chest or body pain. However, these other symptoms were secondary in diagnostic potential to fever characteristic of malaria.

*You would see that you have erema because you would feel hot, and so cold at the same time, weak, and then you wouldn’t even be able to move from here to there.*

*...sometimes the cold is in your bones, you are shivering and weak and you have no power even to stand up.*

In contrast, flu was typically described with respiratory symptoms but lacks the characteristic diagnostic of fever.

*Your nose and throat itches, then I know I have the flu. When I have malaria I have cold, heat, and goosebumps.*

While participants note some differences in symptoms between flu and malaria, others say they are unable to tell the difference on their own. Instead, the only way to tell for sure if they have malaria is to be diagnosed at a clinic or hospital. Many noted that one assumes, and refers to illness, as *otjindjumba*, until tested and diagnosed in a medical setting.

*In the past you would get sick with a disease called erema and otjindjumba. Erema is when you feel hot and cold, otjindjumba is when you have a runny nose. Only when we come to meet doctors with the symptoms which they knew as erema, then the doctors are telling us now that this is malaria. We heard for the first time.*

As a result, while malaria is common to the Kunene, some participants noted they were unsure if they had ever had malaria due to confusion surrounding self-diagnosis.

### Conflicting accounts of aetiology and transmission

Most focus groups report multiple sources of malaria/*erema*. Aetiology typically falls into three groups: environmental/temperature exposure, food-borne, and mosquito-borne. Surprisingly, participants are generally inconsistent about a single source, instead noting that the same disease can result from multiple sources, and that this is not inconsistent with their understanding of the disease. For Herero, less common environmental sources of malaria mentioned also included dirty water and wind. Participants all generally agree that malaria/*erema* is infectious, and transmissible from person-to-person. Lastly, while there was some disagreement, many noted that some cases of malaria are associated with witchcraft, although these are generally only suspected when the infection lingers.

First, most focus groups, both Himba and Herero, explained that becoming too hot or too cold can result in malaria. This can occur, for example, by spending too much time in cold gardens in the morning, by spending the night out in the cold, or by spending extensive time being exposed to heat and sun while cattle herding. For example:

*We go out and sometimes the goats disappear and don’t come home and we have to go back in the bush and look for them. Spend all day in the sun, looking for the goats. When we come back home later you don’t feel very well. The next day you tell your parents “I don’t feel well, what is the problem.” I feel too hot and cold at the same time, and the parents would say “you spend all day in the bush and the sun, maybe this is where you got the malaria from.”*

Additionally, most report varying dietary causes alongside temperature or environmental ones. Consumption of unripe melons and other vegetables was commonly reported as a potential cause. The aetiology of this was explained partially as a rapid shift from a winter diet reliant on sour milk and maize meal porridge to consumption of greens from the garden, but also due to the consumption of melons and other garden-grown crops before they are ripe. For example:

Interviewer*: What causes people to become sick with erema?*

Participant*: The cold. Eating things from the fields like watermelons that are not ripe enough. Also when it’s very hot.*

Interviewer*: When its either very cold or very hot?*

*Participant: Yes when it’s very cold or very hot or when you eat unripe melons from the field.*

Interviewer*: Why do unripe melons give you malaria?*

Participant*: We are not sure but we thought maybe because we spend the time eating other things and suddenly at the rainy season we have all the green things and before they are ready to be consumed we consume them. We thought maybe those are the ones that give disease.*

Lastly, all groups mentioned mosquitoes as a potential source of malaria. In Herero focus groups mosquitoes were the first source raised when queried about the origin of malaria, with environmental and dietary causes being secondary. For Himba, mosquitoes were secondary or were not raised at all by participants. In some groups, it was only when asked directly whether mosquitoes were related to malaria that it was discussed as a potential source. However, Himba note that mosquito bites as the source of malaria was novel information that had been delivered to them by healthcare personnel and public health outreach:

Participant*: My father said before in the past they hear everything from their parents. Now because of the information and knowledge from the doctors, they tell us that malaria comes from mosquitoes.*

Interviewer*: They didn’t know that before?*

Participant*: No we didn’t. We only knew the cold and the heat at the time.*

Others cast doubt on the idea that malaria originates with mosquitoes. One Himba woman, when asked if they believed people get malaria from mosquitoes, was unconvinced, reporting:

*We don’t know what to believe but we hear from educated people. But we stay in our kraals in the bush. We stay there and there are mosquitoes everywhere. We sleep outside and we don’t get malaria. We don’t get sick so why would those mosquitoes give us malaria if we sleep with them and don’t get malaria.*

Importantly, both Himba and Herero participants report that malaria is considered contagious through contact with an infected person. In descriptions of how this process might occur, a respiratory route was typically invoked. For example:

*If I’m sleeping with the other person and I am coughing, that air will go inside the other person, and the other person gets erema. When you have erema you try to be isolated, to say “don’t come closer to me, stay away.”*

The perceived person-to-person transmission of malaria may impact the care of those infected, as participants noted that caregivers may sometimes become ill while caring for a family member with malaria. People noted that if you have malaria you should avoid others, like family members, lest you get them sick also. To avoid breastfeeding infants from getting infected, some recommended keeping them apart from their mothers when they have malaria. Others noted that it was suggested that they should avoid sleeping next to their spouse.

Lastly, we asked if witchcraft was associated with malaria. For both Himba and Herero respondents, this was somewhat controversial. All agreed that malaria was typically not due to witchcraft. However, subset of participants we spoke to, both Himba and Herero, argued that under some circumstances malaria may be the result of witchcraft. This typically occurred when the illness was resistant to treatment, both traditional and biomedical, or when the individual continued to be ill for an extended period. For example:

*The malaria has its time in your body. So if you spend a while with malaria, you used traditional medicines, our herbs, you use the doctors prescriptions and are still stuck with malaria, then you know. We go to get advice from the people with much knowledge and then they will tell us ‘you have been witched’ and we know we have been witched.*

So, while witchcraft may be an atypical cause of illness associated with malaria/*erema*, it can sometimes be attributed under specific circumstances. This is likely due to the general fear of witchcraft in the Kunene writ large, even in more urban settings. As one 50-year-old Herero man who lived near town and had some formal education, put it; “*You can be witched in every aspect of life.*”

### Perception of risk and prevention

Focus groups also included questions and discussion about individual risk factors associated with malaria and seasonal differences associated with infection. Both Himba and Herero describe malaria as a dangerous illness, one that can and has killed children and adults in their community. Many participants believed they have had malaria, some multiple times, although there was some confusion over whether they had experienced malaria or *otjindjumba*/flu.

Participants also generally felt that the risk of malaria was not location specific, and that one was able to contract malaria as readily in town as in the bush. None ascribed any higher risk for infants or children. Instead, participants commented on the variability in an individual’s propensity to become infected as seemingly random, and due to some unknown innate qualities. For example:

*There are people that don’t get malaria at all. There are people that get it once, there are people that even get it twice. People are different. Even the kids like these ones.* [gestures at group of children]* You always have one child that gets sick the most.*

Surprisingly, there was a consensus that malaria risk was highest during the wintertime/dry season. While some noted that one could get malaria during the rainy season, and when there were more mosquitoes (as during the rainy season), the most common answer regarding the seasonal nature of malaria was that the wintertime was the highest risk period for malarial illness. For example, one Himba woman, in expressing doubt that malaria is associated with witchcraft, raised the seasonal nature of malaria, noting:

Participant*: I don’t believe [it’s witchcraft] because we have been told that in some months there will be a disease, and it’s true, there will be a disease.*

Interviewer*: This is true of malaria?*

Participant*: Yes we know that it will come, every now and then. We know in winter definitely we will get sick, we will have malaria. Normally we get sick in wintertime and we know we will have malaria.*

Interviewer*: Not during the rains?*

Participant*: Rarely.*

When questioned about how malaria could be associated with mosquitoes, and yet be more frequent in the dry season when there were rarely mosquitoes, one participant responded:

*Summer is when we get the mosquito bites, and the disease stays in you and manifests in the winter. Manifests and comes to the surface.*

Prevention of malaria is viewed as difficult, if not impossible, by most participants. Herero participants, but not Himba, raised the possibility of mosquito nets to prevent malaria, although only a minority reported actively using them. Rural Himba households sometimes drape mosquito nets over the entrance, acting as a sort of screen door. Herero participants also raised the possibility of spraying for mosquitoes or using storebought creams to prevent mosquito bites as potential methods to avoid malaria. However most instead invoked other non-mosquito related prevention methods, such as avoiding dirty water, avoiding excessive sun exposure, avoiding excessive cold exposure, avoiding others who are ill, and avoiding unripe foods as ways to help prevent malaria. Some participants expressed some exasperation with this line of questioning, noting that it was very difficult to avoid certain foods or activities that might lead to malaria:

*How can we prevent ourselves, when there is food there and you have to go eat? You go eat and then you get sick from it. How can you prevent? There is no prevention.*

### Traditional treatments used as stopgap measures for malaria

Discussions of treatments used for malaria included numerous traditional remedies and preparations. In our focus groups, we recorded at least 20 different types of treatments that may be used to treat malaria, although many were also thought to treat *otjindjumba* and other ailments as well. These treatments run the gamut from steams, teas, and chews to inhalants of various forms. The most common traditional treatments, mentioned by Himba and Herero focus groups, are listed in Table 1.

**Table 1:** Most frequently mentioned traditional treatments for malaria and other ailments

Treatment name	Species	Description
Omandumba	*Pecheul-Loeschea leubnitziae*	Plant boiled and used to create a steam
Omuhama	*Terminalia prunioides*	Tree bark or root boiled in water or milk for tea and/or chewed
Omukange	*Cammiphora pyracanthoides*	Tree bark steeped in water and drank
Otjipumba	*Loxodonta africana* dung	Dried elephant dung, steeped in water or boiled as a tea and drank, or burned and the smoke inhaled

Steams, such as *omandumba*, are among the most common treatments for malaria as well as other respiratory ailments including *otjindjumba*. Traditional preparation of steams is a process whereby particular plants are boiled and then hot rocks added from the fire. The patient then covers themselves with a blanket over the steaming water to inhale the steam.

*If you see that you are still struggling, you go get omandumba for steam. Go get omandumba, chop it into little pieces, and put it in pot with water. While its boiling, get a stone to put in the fire. That stone burns and becomes red. After the omandumba water is boiled, that stone is completely red, you pick up the red stone, you put in the water, and you cover yourself with a blanket, then you inhale, you steam yourself. That water from your body will get out, and will release all the sickness.*

Numerous types of teas prepared from tree bark, leaves, and plant roots were also described. The most important of these is *otjipumba* or elephant dung. This is typically made into a tea which is described as a powerful curative treatment for malaria and other illnesses. Another common preparation is to instead burn the dried dung and inhale the smoke, although this was typically described as a treatment for headaches and congestion. Participants explained that because the local desert elephants eat all different types of plants, including all the ones used for traditional treatments, the dung is a sort of concentrated wonder drug that combines all these different medicines. Anecdotally, Himba in the area describe elephant-dung based traditional medicines as essential treatment, allowing them to survive and make it to the hospital to receive biomedical treatment if needed. However, there are few elephants in the area whereby dung could be collected. Participants note that they typically collect it when traveling and store it for later use. Others note that they must travel to Sesfontein, towards the southern boundary of where Himba traditionally reside, and nearly 150 km from the regional town centre, to collect it.

**Fig. 1:**
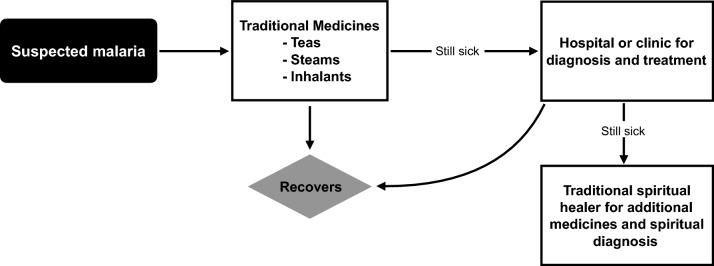
Flowchart of typical malaria treatment for Himba

Traditional treatments were described by both Himba and Herero as the first line of defense for illness, including malaria. However, should traditional treatments not be effective, then the next course of action is to seek assistance at the local clinic or the regional hospital in Opuwo (see Fig. 1). Pursuing clinic or hospital treatment for these illness was considered normative, although some expressed distrust of doctors and nurses (see below). When deciding when to seek further treatment, above and beyond traditional medicines, Himba and Herero participants typically describe inadequate response to traditional medicines or advancing symptoms as signs that biomedical treatment is needed. For example:

*Once you feel that their bones are getting weak, that’s when you decide to go to the hospital. Before you get weak you will first use the traditional medicine, the ones that can cure malaria, but when you come to the point that you got weak, that’s when you decide to go to the hospital.*

### Traditional healers used in extenuating circumstances

In the Kunene, traditional spiritual healers, sometimes referred to witchdoctors, are called *otjimbanda*. Some Himba and Herero participants noted that they would generally not go to the *otjimbanda* for malaria, that instead this was a disease that could be treated at home or in the hospital. As one participant put it; *“…in the world of today there is no otjimbanda that cures malaria.*”

It was also pointed out that traditional medicines are available everywhere. For the most part, specialized knowledge of traditional medicine was not needed to produce traditional treatments like steams and teas. Although there was some variability across focus groups in the types of use for traditional treatments, focus group participants explained that these traditional medicines were known of and available to anyone. As a result, the *otjimbanda* could not offer additional traditional medicines beyond what the general Himba or Herero already knows, making them less useful in the case of malaria. As one participant described:

*And we also realize that when we go to the otjimbanda he also treats you with the medicine that you already know, that you use, so it doesn’t make any sense.*

However, extenuating illness may be blamed on witchcraft. Lingering illness, long-lasting symptoms without an obvious cause, or failure of prior traditional and/or biomedical treatments may be suspected to be the result of witchcraft. While not true of all participants, many noted that should they not recover using medicines from the hospital or clinic, then they would seek additional assistance from traditional healers. As one participant described:

*We don’t go to the otjimbanda because we know that’s not a disease to go to the otjimbanda for, unless a boy has had malaria and they treat the boy and after that the boy is still weak for a long time and they say this might be something else. That is maybe when they will seek out an otjimbanda.*

### Medical mistrust

Seeking treatment at a hospital or clinic following use of traditional medicines was considered normative by Himba and Herero. Participants easily recounted the procedure of testing associated with a suspected malaria case at the hospital, and the treatments provided. However, a subset of participants expressed dissatisfaction with clinic or hospital treatment. For example, as one elderly Himba man recounted:

*I only trust my traditional medicine... I don’t trust the hospital medicine. When we used to go to the hospital, maybe I introduce myself to the doctor. I’ve got disease, I’ve got flu or malaria. The doctor gives me paracetamol. I give up on that medicine because any time I get any disease, they give me paracetamol, the white pill. I give up, it’s better to use my own traditional medicine. I’m tired of paracetamol, I take it but I didn’t even get better from this medicine. That’s why I trust the traditional medicine instead of the hospital.*

Others report similar complaints that they do not recover, or do not recover quickly enough using biomedical treatments at hospitals and clinics. For this subset of Himba and Herero, they instead expressed that traditional medicine was superior to the medicines provided in a hospital setting. In some cases, this mistrust drove individuals to avoid biomedical treatment when sick, or to travel to a hospital outside of the Kunene where they felt they would be better treated.

## Discussion

In this study, local explanatory models of malaria were explored in two groups of Kunene pastoralists where there is currently little written on cultural models of disease. These results yield important interpretations, gaps in knowledge, and misunderstandings that may hinder the ability to test, treat, and ultimately eradicate the disease in Namibia. Focus group discussions suggested dissonance regarding interpretations of influenza and malaria, which may impact testing and treatment of malaria. As Namibia attempts to lower locally transmitted cases, particularly in rural communities that frequent the border with Angola, more specialized approaches and interventions may be necessary. These findings are interpreted with an eye towards applying results in future public health initiatives and information campaigns.

Results illustrate significant deficits in knowledge about malaria in these communities, although the more formally educated and market-integrated Herero tend to have more knowledge than Himba. First, focus groups in this study yielded a striking finding that Himba and Herero both have difficulty in differentiating between influenza (or *otjijumba*) and malaria (or *erema*). The cultural category of *otjijumba* appears to be relatively broad, encompassing respiratory illnesses and other diseases, including those with fever. The consensus was that an illness was assumed to be *otjijumba* unless diagnosed as malaria by a doctor. However, participants recognized that malaria is typically associated with severe fatigue and fever. This aetiological confusion may explain beliefs regarding respiratory transmission of malaria. Other African populations have multiple, and sometimes conflicting, disease categories related to malaria, fever, or other types of illnesses. For example, the Bwatiye of Nigeria classify febrile illnesses that are more benign into “female” malaria, and illnesses that take longer to recover from as “male” malaria [36]. Similar to other groups [37], Himba and Herero also understand malaria to be a “new” illness, which may explain lack of a more cohesive and independent illness category. In this case, the initial assumption of *otjindjumba*, combined with the inability to self-diagnose malaria, may result in delayed testing and treatment in a clinic or hospital setting.

Most surprisingly was the widespread belief that risk of malaria is highest during the winter or dry season, when mosquitoes are infrequent, and not during the rainy season. Malaria cases typically peak between January and April (during the rainy season) before falling to a nadir in June and July (during the dry season), and maintaining low levels through December [38]. It is unclear what the source of this discrepancy is, but this may suggest that some of the dry season/winter illness assumed to be malaria could be other respiratory diseases (like influenza), while a portion of the wet season/summer illnesses assumed to be *otjindjumba* are more likely to be malaria. There is a well-established relationship between low temperature and humidity and respiratory infections [39]. This pattern has been found in informal settlements in Namibia, where low temperature corresponds to peaks in common colds, assorted respiratory infections, and pneumonia [40]. Northern Namibia is subject to climatic anomalies, including periods of rain and drought, and increased rainfall historically corresponds to higher malaria infection rates [41]. It is possible that the confusion and overlap in illness categories of malaria and *otjindjumba* obscures local interpretations of the seasonal rhythms of malaria and respiratory infections. This could be particularly problematic when interpretation of illness, and in particular assumption of *otjindjumba* in the wet season, delays testing and treatment.

Considerable variety in interpretations of the aetiology of malaria was found, including dietary, environmental, and mosquito-borne origins. Himba are less likely to identify mosquitoes as the source of infection, casting doubt on this aetiology and instead emphasizing other sources of illness. The most common of these were dietary causes and heat/cold exposure. Conversely Herero view mosquitoes as the main, but not only source of malaria. This discrepancy, perhaps based on higher rates of formal education in Herero, is similar to educational gaps found in other populations [42]. Relatedly, Herero, but not Himba, were more likely to raise mosquito-bite preventing interventions, such as mosquito nets or IRS. Both groups view malaria as a “natural” illness, which may come with a different set of expectations regarding treatment and support [43, 44]. The febrile nature of malaria symptomology makes assumptions of temperature-based aetiology common, and other studies of local explanatory models in other populations have documented temperature or sun exposure as a perceived cause of malaria [42, 45–49]. Here, the aetiology may undermine perceptions self-efficacy for Himba and Herero, who tend to view temperature-based or food-based origins of malaria as unavoidable, negatively impacting behavioral change leading to disease prevention [13, 50]

Traditional medicine use is common in the study area, and traditional medicines were the first line of defense for any illness. As detailed by previous work, Kunene pastoralists have a significant library of traditional medicines to draw from [51–53], and a number of these plants have been found to have anti-malarial properties [54]. Both Himba and Herero were able to name and identify several traditional treatments used for cases of *otjindjumba* or malaria, and these treatments were used first, at home, prior to seeking medical treatment. However, use of traditional spiritual healer is less frequent, and typically only used after treatment at a clinic or hospital has failed. This suggests that local spiritual practitioners aren’t hampering biomedical treatment, and, like other populations, that local models of malaria may co-exist with witchcraft models of illness [49]. The reliance on numerous traditional medicines may hinder early testing and treatment, as participants described that they would attempt these first, and then only seek treatment at the clinic or hospital if the illness persisted or became worse. Delays associated with a reliance on traditional remedies appear to be common across many populations [55, 56], but in other cases traditional spiritual beliefs and practices may not impede and actually support biomedical treatment [49, 57]. For Himba and Herero, delay is likely exacerbated by the inability to distinguish between respiratory illnesses and malaria, and malaria is likely only diagnosed in a healthcare setting after traditional treatments have failed and the individual’s health has continued to decline. Such delays make controlling and ultimately eradicating malaria more difficult [9].

While seeking biomedical treatment for malaria at clinics and hospitals is common and normative for Himba and Herero, a subset of participants in our focus groups raised issues of poor treatment, inadequate care, and lack of trust in healthcare providers and in healthcare settings. This topic was not formally studied as part of this project, nor were issues of medical mistrust included in the focus group question prompts. However, previous work in Himba communities in the Kunene finds that indices of medical mistrust map onto negative health experiences, and are driven by perceived maltreatment and discrimination from healthcare personnel [27]. Additionally, medical mistrust in Himba and Herero communities is associated with vaccine interest and beliefs about safety [58]. Elsewhere in Namibia, structural factors such as long wait times and distance and cost of transport to hospitals and clinics, coupled with negative experiences with healthcare personnel can act to undermine care [59, 60]. Similar issues are common across Africa, and undermine perceptions of public health services [61]. As medical mistrust is typically linked to underutilization of healthcare services [62], these issues may continue to impede work on malaria reduction and eradication in Africa, although there is still little quantitative data on the role of medical mistrust in low- and middle-income countries [63].

The results of this study point to a strong need for additional educational and public health outreach campaigns, particularly for rural Kunene residents who lack formal education. Misunderstandings about the aetiology and seasonal nature of malaria, differentiation from respiratory infections, and education on additional modes of prevention are sorely needed. This should include detailed symptomology to help Kunene residents decide when to seek healthcare for potential malaria, and when to wait out a mild respiratory infection. Such knowledge could theoretically reduce the time to diagnosis and treatment in a clinic or hospital setting, particularly if residents are less likely to rely on traditional remedies until the illness worsens. Better local understanding of aetiology may increase perceptions of self-efficacy which can itself lead to disease prevention behaviours [50]. Outreach and intervention efforts may benefit from the application of local terms to help distinguish diseases in a way that is more culturally relevant and locally salient [64]. Public health outreach and educational initiatives may be most likely to succeed when they incorporate locally prestigious figures, such as village chiefs [65]. Incorporating local leaders has the added advantage of being able to relay concepts in culturally relevant terms, and unlike health practitioners or government officials, not being subjects of medical mistrust. Across much of Africa, traditional leaders maintain high levels of trust, particularly in rural populations [66]. Previous work in the Kunene indicates that both doctors and local chiefs are viewed as influential in vaccine decision making, although doctors were favoured in more urban settings while local chiefs were favoured in rural ones [67]. Initiatives and outreach which are both appropriate and “culturally compelling” are likely to be more successful [13].

## Conclusions

Structural issues in the healthcare system, poverty, and the proportion of individuals living in rural and remote areas can negatively impact testing and treatment for malaria. Understanding local cultural models of disease and norms of treatment are crucial in designing healthcare services and outreach. As Namibia shrinks the number of locally contracted malaria cases in pursuit of eradication, targeting at-risk indigenous groups near the Angolan border with bespoke epidemiological strategies and outreach will become increasingly important [68]. Designing such outreach requires an understanding of how malaria is understood by these communities and how treatment seeking is enacted. Results from this study indicate there are significant cultural hurdles towards early testing and treatment in indigenous Himba and Herero communities, including difficulty differentiating malaria from respiratory diseases, little understanding of the aetiology, seasonality, or methods of prevention of malaria, and reliance on traditional medicines that may delay seeking healthcare. This suggests that these groups could benefit from targeted informational and educational campaigns focused on malaria. Future outreach efforts should seek to incorporate cultural models of disease in addressing knowledge deficits.

## Data Availability

Data are available from the corresponding author upon reasonable request.
